# Quantitative analysis of rutin, quercetin, naringenin, and gallic acid by validated RP- and NP-HPTLC methods for quality control of anti-HBV active extract of *Guiera senegalensis*

**DOI:** 10.1080/13880209.2017.1300175

**Published:** 2017-03-10

**Authors:** Perwez Alam, Mohammad K. Parvez, Ahmed H. Arbab, Mohammed S. Al-Dosari

**Affiliations:** a Department of Pharmacognosy, College of Pharmacy, King Saud University, Riyadh, Saudi Arabia;; b Department of Pharmacognosy, College of Pharmacy, Omdurman Islamic University, Khartoum, Sudan

**Keywords:** Natural products, combretaceae, plant extract, antiviral, flavonoids, polyphenols

## Abstract

**Context:***Guiera senegalensis* J.F. Gmel (Combretaceae) is a folk medicinal plant used in various metabolic and infectious diseases. In addition to its antiviral activities against herpes and fowlpox, the anti-HBV efficacy is very recently reported.

**Objective:** To develop and validate simple, sensitive RP-/NP-HPTLC methods for quantitative determination of biomarkers rutin, quercetin, naringenin, and gallic acid in the anti-HBV active *G. senegalensis* leaves ethanol-extract.

**Materials and methods:** RP-HPTLC (rutin & quercetin; phase- acetonitrile:water, 4:6) and NP-HPTLC (naringenin & gallic acid; phase- toluene:ethyl acetate:formic acid, 6:4:0.8) were performed on glass-backed silica gel plates 60F_254_-RP18 and 60F_254_, respectively. The methods were validated according to the ICH guidelines.

**Results:** Well-separated and compact spots (R_f_) of rutin (0.52 ± 0.006), quercetin (0.23 ± 0.005), naringenin (0.56 ± 0.009) and gallic acid (0.28 ± 0.006) were detected. The regression equations (Y) were 12.434x + 443.49, 10.08x + 216.85, 11.253x + 973.52 and 11.082x + 446.41 whereas the coefficient correlations (*r*^2^) were 0.997 ± 0.0004, 0.9982 ± 0.0001, 0.9974 ± 0.0004 and 0.9981 ± 0.0001, respectively. The linearity ranges (ng/spot) were 200–1400 (RP-HPTLC) and 100–1200 (NP-HPTLC). The LOD/LOQ (ng/band) were 33.03/100.1 (rutin), 9.67/29.31 (quercetin), 35.574/107.8 (naringenin), and 12.32/37.35 (gallic acid). Gallic acid (7.01 μg/mg) was the most abundant biomarker compared to rutin (2.42 μg/mg), quercetin (1.53 μg/mg) and naringenin (0.14 μg/mg) in the extract.

**Conclusion:** The validated NP-/RP-HPTLC methods were simple, accurate, and sensitive for separating and quantifying antiviral biomarkers in *G. senegalensis*, and endorsed its anti-HBV activity. The developed methods could be further employed in the standardization and quality-control of herbal formulations.

## Introduction

High-performance thin-layer chromatography (HPTLC) has recently become a conventional analytical tool for the quality-control of herbal drugs because of its low operation-cost, high sample-throughput and need for minimum sample clean-up (Alam et al. 2014). With HPTLC, qualitative and quantitative analyzes of multiple compounds can be done simultaneously by using small volume of mobile phase (Faiyazuddin et al. 2010). The developed HPTLC chromatograms are useful in identification of biomarkers in various herbal formulations by comparing the fingerprints with standards (Siddiqui et al. 2014). It is widely employed for the identification, purity testing, stability, dissolution or content uniformity of crude extracts of plant and animal origin, fermentation mix, drugs and excipients, including pharmaceutical, cosmetic and nutrient formulations (Alajmi et al. 2013).

*Guiera senegalensis* J.F. Gmel (Combretaceae) is one of the popular African folk medicine plants for treating a wide range of metabolic and infectious diseases (Bosisio et al. 1997; Somboro et al. 2011; Suleiman 2015). The dried bitter leaves are the most important part of the plant, commonly sold in African markets as ‘Cure all’ medicine. An antitussive sirup (Nger), prepared from the *G. senegalensis* leaves, has been commercialized in Senegal (Sanogo et al. 1998). Decoctions and various preparations of *G. senegalensis* are used to treat sexually transmitted, gastrointestinal, respiratory, fungal, bacterial and malarial diseases (Bosisio et al. 1997; Abubakar et al. 2000; Silva & Gomes 2003; Somboro et al. 2011; Akuodor et al. 2013; Suleiman 2015). Moreover, the plant extract has shown to have antioxidative (Bouchet & Barrier 1998), anti-inflammatory (Sombié et al. 2011) and acaricidal (Osman et al. 2014) activities. Moreover, *G*. *senegalensis* was also reported to have antiviral activities against fowl pox (Lamien et al. 2005) and herpes (Silva et al. 1997) infections. Very recently, we demonstrated *in vitro* anti-hepatitis B virus (HBV) efficacy of *G. senegalensis* leaves extract (Parvez et al. 2016). Further, among several groups of phytoconstituents, four flavonoids (catechin, myricitrin, rutin, and quercetin) (Bucar et al. 1996; Ficarra et al. 1997; Males et al. 1998), two alkaloids (harman and tetrahydroharman or eleagnine) and one naphthyl butenone (guieranone A) are identified in the plant (Combier et al. 1997; Mahmoud & Sami 1997; Fiot et al. 2006).

Recently, quercetin, a flavonoid is reported for its anti-HBV potential *in vitro* (Cheng et al. 2015). Although biomarkers quercetin and rutin have been identified in *G. senegalensis* by HPLC (Males et al. 1998), a complete validated HPTLC method has not been reported yet for their quantitative analysis in *G. senegalensis*. Therefore, the present study intended to develop and validate normal phase (NP)- and reverse phase (RP)-HPTLC methods for quantifying the contents of rutin, quercetin, naringenin and gallic acid in the anti-HBV active extract of *G. senegalensis* leaves.

## Materials and methods

### Plant material

Leaves of *G. senegalensis* locally known as ‘Gubeish’ were collected in March, 2015 from Kordofan state, Sudan. The plant material was authenticated by Prof. Ismail Mirghani, a taxonomist at the Forestry Research Center (FRC), Khartoum, Sudan, where a voucher specimen (No. 891) was deposited. Further authentication was confirmed at the herbarium of College of Pharmacy, King Saud University, Saudi Arabia.

### Preparation of G. senegalensis leaves ethanol-extract (GSEE)

The leaves were shade dried at room temperature for 8 days. The dried leaves (50 g) were ground to fine powder using mortar-pestle and extracted with 500 mL of 70% ethanol (Merck) for 24 h with intermittent shaking. The extraction process was repeated two times with fresh solvent. Then, extracts were pooled, filtered (Whatmann filter paper No. 1) and dried under reduced pressure using rotary evaporator (R-210, BUCHI).

### Apparatus and reagents

The biomarkers (rutin, quercetin, naringenin and gallic acid) were procured from Sigma Aldrich (USA). While AR grade chemicals *viz*., ethanol, acetonitrile, toluene, ethyl acetate and formic acid were procured from BDH (UK), HPLC grade ethanol and methanol were procured form Merk (Germany). For the analysis of samples and standards, glass-backed silica gel 60F_254_ RP-18 plate (for RP-HPTLC) and glass-backed silica gel 60F_254_ plate (for NP-HPTLC) were purchased from Merck (Germany). CAMAG Automatic TLC Sampler-4 (Switzerland) was used to apply the biomarkers and GSEE, band wise to the chromatographic plates and development was accomplished in automatic development chamber (ADC2) (Switzerland). The developed HPTLC Plates were then documented by CAMAG TLC Reprostar 3 and scanned by CAMAG CATS 4 (Switzerland).

### HPTLC instrumentation and conditions

The HPTLC analysis of the biomarkers in GSEE was carried out on NP and RP-HPTLC plates (10 × 10 cm) where the band size of each track was 6 mm wide and 8 mm apart. The samples were applied on the HPTLC plates (160 nL/s). The plates were developed in pre-saturated twin-trough glass chamber (20 × 10 cm) at room temperature (25 ± 2 °C) and humidity (60 ± 5%) using acetonitrile and water (4:6, v/v) for RP-HPTLC, and toluene, ethyl acetate and formic acid (6:4:0.8, v/v/v) for NP-HPTLC analysis. The developed and dried RP-HPTLC and NP-HPTLC plates were quantitatively analyzed at 360 and 275 nm in absorbance mode, respectively.

### Preparation of standard stock solutions

Standard stocks of rutin, quercetin, naringenin and gallic acid were prepared in methanol (1 mg/mL). The stocks of rutin and quercetin were further diluted to furnish different concentrations ranging from 10 to 140 μg/mL. All the dilutions (10 μL, each) were applied through microliter syringe attached with the applicator on the RP-HPTLC plate to furnish the linearity range of 100-1400 ng/band for rutin and quercetin. Similarly, the dilutions of naringenin and gallic acid ranging from 10 to 120 μg/mL (10 μL, each) were applied to NP-HPTLC plate to furnish the linearity range of 100–1200 ng/band.

### Method validation

Method of validation was carried out as per International Conference on Harmonization (ICH) guidelines for linearity range, limit of detection (LOD), limit of quantification (LOQ), precision, recovery as accuracy and robustness (ICH 2005). The determination of LOD and LOQ was calculated using formula *LOD = 3.3(SD/S)* and *LOQ = 10(SD/S)*, respectively, based on the standard deviation of the response (SD) and the slope (S) of the calibration curve. The precision (Intra-day and Inter-day) of the proposed HPTLC methods were evaluated for all biomarkers by performing replicate analysis (*n* = 6) at three different concentration levels (low, medium and high) *viz.* 400, 600 and 800 ng/band. The precision was recorded as Mean ± SD, %RSD and SEM of each calibration level. Recovery as accuracy studies involved the addition of a known amount of analyte to a sample, and determining the percentage of added analyte. For the biomarkers rutin, quercetin, naringenin and gallic acid, a known amount of 50, 100 and 150% of 200 ng, each was added and the recovery percentage of the spiked standards was estimated. The robustness of the proposed HPTLC methods were performed to analyze its capacity to remain unaffected by a small, but deliberate variations in mobile phase composition, mobile phase volume used for saturation and duration of saturation which indicates the reliability of the method during normal use. The robustness study was performed in replicate analysis (*n* = 6) for all the markers at 300 ng/band concentration. The results were evaluated in terms of SD, %RSD and SEM of peak area. In RP-HPTLC method, the mobile phases were prepared from acetonitrile: water (4:6, v/v) in different proportions (3.8:6.2, v/v and 4.2:5.8, v/v) and analyzed. In case of NP-HPTLC method, the different mobile phases (5.8:4.2:0.8 and 6.2:3.8:0.8, v/v/v) were prepared from toluene: ethyl acetate: gallic acid (6:4:0.8, v/v/v) and used for the analysis of markers to check its robustness. In addition to the minor variations in the mobile phases, the volume used for saturation was also varied from 20 to 18 and 22 mL. The duration of saturation also varied to 10 and 30 min from 20 min in the analysis.

### Statistical analysis

Results were expressed as mean ± SD. Total variation present in a set of data was estimated by one-way analysis of variance (ANOVA) followed by Dunnet’s test. *p <* 0.01 was considered significant.

## Results

### Method development

**Figure 1.**
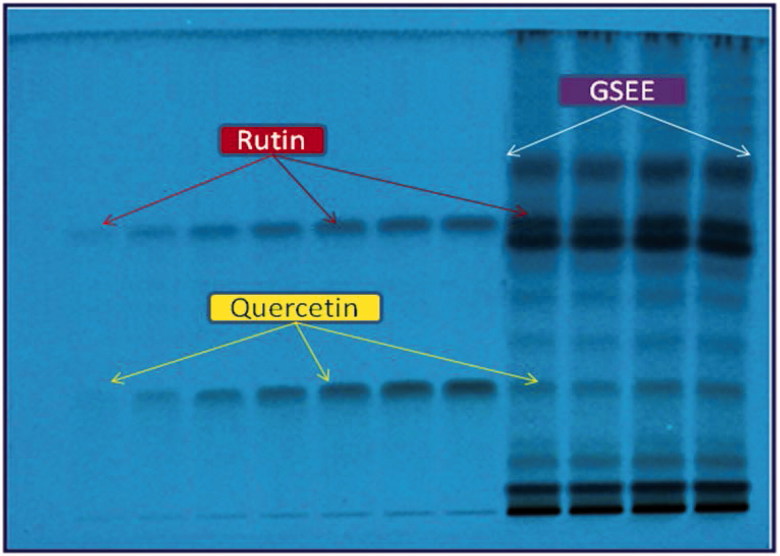
Pictogram of developed RP-HPTLC plate at *λ* = 254 nm; mobile phase, acetonitrile:water (4:6, v/v).

**Figure 2.**
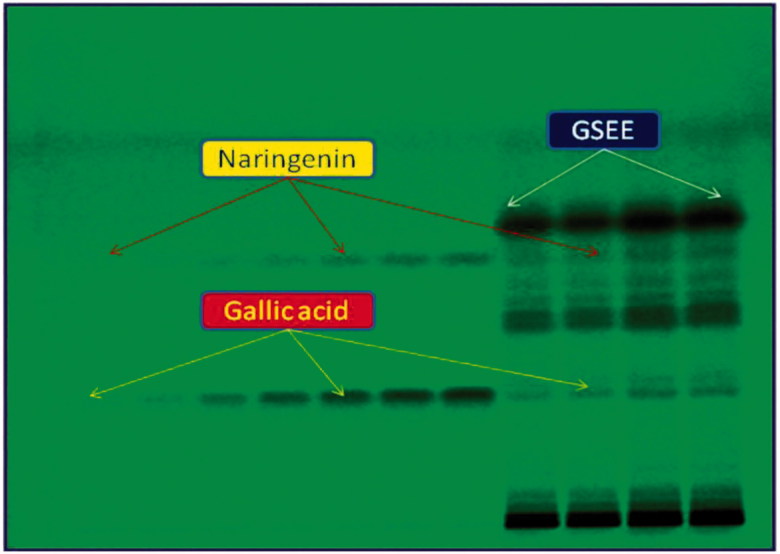
Pictogram of developed NP-HPTLC plate at *λ* = 254 nm; mobile phase, toluene:ethyl acetate:formic acid (6:4:0.8, v/v/v).

The mobile phase used in RP- and NP-HPTLC analyses was selected by testing several compositions of different solvents. Of these, combination of acetonitrile and water (4:6, v/v) under chamber saturation condition was found to be the best mobile phase for the development and quantitative analysis of rutin and quercetin on RP-HPTLC plates. This method exhibited the clear separation of the two biomarkers along with the different constituents of GSEE (Figure 1). On the other hand, for the analysis of naringenin and gallic acid on NP-HPTLC plates, the best mobile phase was the combination of toluene, ethyl acetate and gallic acid (6:4:0.8, v/v/v) which allowed their clear separation along with the different constituents of GSEE (Figure 2). The optimized saturation time and volume of mobile phase for saturation were 20 min and 20 mL, respectively.

**Figure 3.**
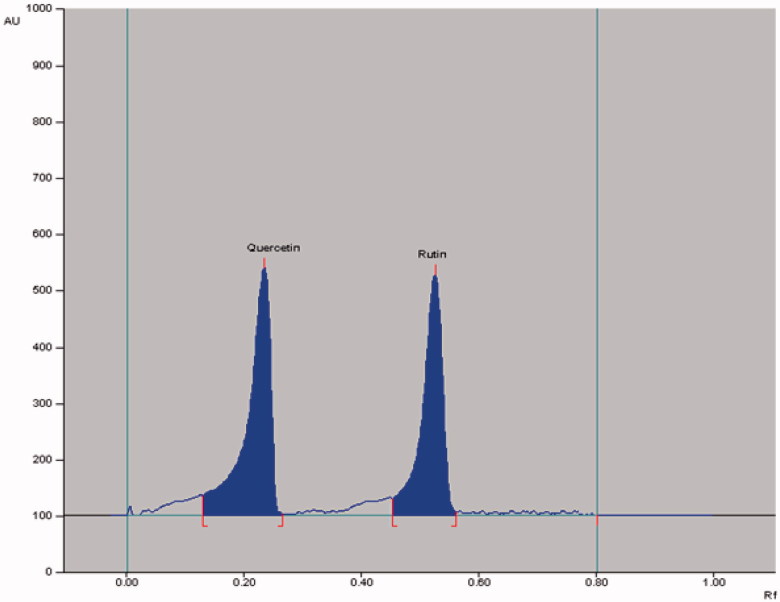
Chromatogram of biomarkers quercetin (R_f_ = 0.23; 800 ng/spot) and rutin (R_f_ = 0.52; 800 ng/spot) at *λ* = 360 nm; mobile phase, acetonitrile:water (4:6, v/v).

**Figure 4.**
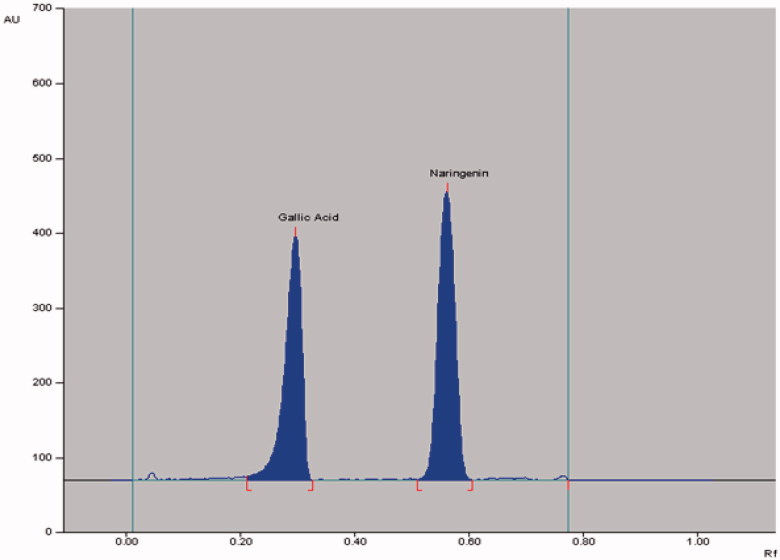
Chromatogram of biomarkers gallic acid (R_f_ = 0.28; 600 ng/spot) and naringenin (R_f_ = 0.55; 600 ng/spot) at 275 nm; mobile phase, toluene:ethyl acetate:formic acid (6:4:0.8, v/v/v).

The densitometric analysis of the biomarkers by the two HPTLC methods showed clearly separated compact, sharp, symmetrical and high resolution bands of rutin, quercetin, naringenin and gallic acid. While the bands of rutin and quercetin were obtained at R_f_ 0.52 ± 0.006 and 0.23 ± 0.005, respectively (Figure 3), those of naringenin and gallic acid were recorded at R_f_ 0.56 ± 0.009 and 0.28 ± 0.006, respectively (Figure 4). The developed methods were thus, found quite selective with a good baseline resolution.

### Method validation

Linearity of marker compounds rutin, quercetin, naringenin and gallic acid were validated by the linear regression equation and correlation coefficient. The seven-point calibration curve for rutin and quercetin was found linear in the range of 200–1400 ng whereas for naringenin and gallic acid it was in the range of 100–1200 ng. The observed regression equation (Y) and coefficient correlation (*r*^2^) values for the biomarkers (Table 1) revealed a good linearity response for the developed methods. The LOD and LOQ for rutin, quercetin, naringenin, and gallic acid were also recorded (Table 1) which indicated that the proposed method exhibits a good sensitivity for the simultaneous quantification of the above compounds. The %recovery, %RSD, and SEM were recorded in for rutin and quercetin (Table 2), and naringenin and gallic acid (Table 3) for recoveries as accuracy study for the proposed methods. The intra- and inter-day precision (*n* = 6) for the proposed RP- and NP-HPTLC methods were recorded as %RSD and SEM for rutin and quercetin (Table 4), and for naringenin and gallic acid (Table 5). The observed low values of %RSD and SEM indicated the good precision of both methods. Further, the low values of SD, %RSD and SEM obtained after introducing small deliberate changes in the two methods demonstrated the robustness of NP-HPTLC for rutin and quercetin (Table 6), and RP-HPTLC for naringenin and gallic acid (Table 7).

**Table 1.** R_f_, Linear regression data for the calibration curve of rutin, quercetin, naringenin and gallic acid (*n* = 6).

Parameters	Rutin	Quercetin	Naringenin	Gallic acid
Linearity range (ng/spot)	200–1400	200–1400	100–1200	100–1200
Regression equation	*Y* = 12.434*x* + 443.49	*Y* = 10.08*x* + 216.85	*Y* = 11.253*x* + 973.52	*Y* = 11.082*x* + 446.41
Correlation (*r*^2^) coefficient	0.997 ± 0.0004	0.9982 ± 0.0001	0.9974 ± 0.0004	0.9981 ± 0.0001
Slope ± SD	12.434 ± 0.124	10.08 ± 0.029	11.253 ± 0.121	11.082 ± 0.041
Intercept ± SD	443.49 ± 11.547	216.85 ± 8.171	973.52 ± 12.301	446.41 ± 15.557
Standard error of slope	0.050	00.012	0.049	0.016
Standard error of intercept	4.713	3.335	5.021	6.349
R_f_	0.52 ± 0.006	0.23 ± 0.005	0.56 ± 0.009	0.28 ± 0.006
LOD	33.03 ng band^−1^	9.67 ng band^−1^	35.574 ng band^−1^	12.32 ng band^−1^
LOQ	100.1 ng band^−1^	29.31ng band^−1^	107.8 ng band^−1^	37.35ng band^−1^

**Table 2.** Recovery as accuracy studies of the proposed RP-HPTLC method of rutin and quercetin (*n* = 3).

	Theoretical concentration of rutin (ng/mL)	Concentration of rutin found (ng/mL) *±* SD	%RSD	SEM	% Recovery
Percent (%) of rutin added to analyte					
0	200	197.48*±*1.54	0.781	0.629	98.74
50	300	297.09*±*1.75	0.590	0.715	99.03
100	400	396.55*±*2.92	0.737	1.194	99.13
150	500	498.06*±*2.96	0.595	1.211	99.61
Percent (%) of quercetin added to analyte					
0	200	200.58*±*2.11	0.743	0.608	100.29
50	300	296.03*±*3.46	0.940	1.136	98.67
100	400	404.43*±*5.18	0.845	1.395	101.10
150	500	498.04*±*6.99	0.798	1.624	99.60

**Table 3.** Recovery as accuracy studies of the proposed NP-HPTLC method of naringenin and gallic acid (*n* = 3).

	Theoretical concentration of naringenin (ng/mL)	Concentration of naringenin found (ng/mL) *±* SD	%RSD	SEM	% Recovery
Percent (%) of naringenin added to analyte					
0	200	198.43*±*2.66	1.343	1.088	99.21
50	300	297.37*±*3.59	1.207	1.465	99.12
100	400	398.09*±*5.91	1.486	2.415	99.52
150	500	499.27*±*7.55	1.513	3.084	99.85
Percent (%) of gallic acid added to analyte					
0	200	197.18*±*2.11	1.073	0.863	98.59
50	300	298.06*±*3.46	1.162	1.413	99.35
100	400	397.12*±*5.18	1.305	2.115	99.28
150	500	499.62*±*6.99	1.399	2.854	99.92

**Table 4.** Precision of the proposed RP-HPTLC method of rutin and quercetin (*n* = 3).

	Intra-day precision			Inter-day precision		
	Average Conc. found ± SD	%RSD	SEM	Average Conc. found ± SD	%RSD	SEM
Conc. of rutin (ng/spot)						
400	398.16*±*1.55	0.390	0.632	394.14*±*1.49	0.379	0.610
600	600.80*±*3.22	0.536	1.315	595.21*±*3.01	0.506	1.230
800	797.95*±*5.63	0.706	2.299	792.40*±*5.21	0.657	2.127
Conc. of quercetin (ng/spot)						
400	397.76*±*2.31	0.581	0.094	395.58*±*2.19	0.554	0.895
600	600.34*±*3.59	0.599	1.468	597.36*±*3.26	0.546	1.334
800	799.82*±*2.82	0.353	1.153	794.27*±*2.51	0.317	1.028

**Table 5.** Precision of the proposed NP-HPTLC method of naringenin and gallic acid (*n* = 3).

	Intra-day precision			Inter-day precision		
	Average Conc. found ± SD	%RSD	SEM	Average Conc. found ± SD	%RSD	SEM
Conc. of naringenin (ng/spot)						
400	398.09*±*3.51	0.883	1.435	395.69*±*3.10	0.784	1.266
600	597.02*±*5.13	0.926	2.258	594.71*±*5.25	0.883	2.145
800	801.52*±*7.92	0.988	3.233	798.85*±*7.37	0.923	3.011
Conc. of gallic acid (ng/spot)						
400	398.53*±*2.34	0.587	0.956	395.82*±*2.21	0.554	0.903
600	597.24*±*5.86	0.981	2.392	595.44*±*5.13	0.546	2.096
800	797.30*±*7.09	0.890	2.896	791.88*±*6.98	0.317	2.851

**Table 6.** Robustness of the proposed RP-HPTLC method (*n* = 3).

	Rutin (300 ng/band)			Quercetin (300 ng/band)		
Optimization condition	SD	%RSD	SEM	SD	%RSD	SEM
Mobile phase composition; (Acetonitrile: water)						
(4:6)	2.154	0.543	0.879	3.641	0.914	1.486
(3.8:6.2)	2.235	0.562	0.912	3.459	0.869	1.411
(4.2:5.8)	1.914	0.484	0.781	3.215	0.810	1.312
Mobile phase volume (for saturation)						
(18 mL)	2.112	0.533	0.862	3.925	0.981	1.602
(20 mL)	2.214	0.558	0.903	3.858	0.973	1.575
(22 mL)	2.175	0.548	0.889	3.815	0.960	1.557
Duration of saturation						
(10 min)	2.231	0.563	0.911	3.835	0.967	1.565
(20 min)	2.262	0.571	0.923	3.792	0.954	1.547
(30 min)	2.218	0.561	0.905	3.866	0.966	1.578

**Table 7.** Robustness of the proposed NP-HPTLC method (*n* = 3).

	Naringenin (300 ng/band)			Gallic Acid (300 ng/band)		
Optimization condition	SD	%RSD	SEM	SD	%RSD	SEM
Mobile phase composition; (Toluene: ethyl acetate: formic acid)						
(6:4:0.8)	4.151	1.394	1.695	3.133	1.044	1.278
(5.8:4.2:0.8)	3.963	1.338	1.617	3.395	1.129	1.385
(6.2:3.8:0.8)	4.514	1.509	1.842	3.744	1.249	1.528
Mobile phase volume (for saturation)						
(18 mL)	4.243	1.424	1.732	3.417	1.139	1.394
(20 mL)	4.157	1.404	1.696	3.614	1.202	1.475
(22 mL)	4.323	1.445	1.764	3.442	1.149	1.405
Duration of saturation						
(10 min)	4.212	1.414	1.719	3.442	1.147	1.405
(20 min)	4.146	1.401	1.692	3.413	1.135	1.393
(30 min)	4.293	1.435	1.752	3.435	1.146	1.403

### Application of the NP- and RP-HPTLC for the analysis of biomarkers in GSEE

**Figure 5.**
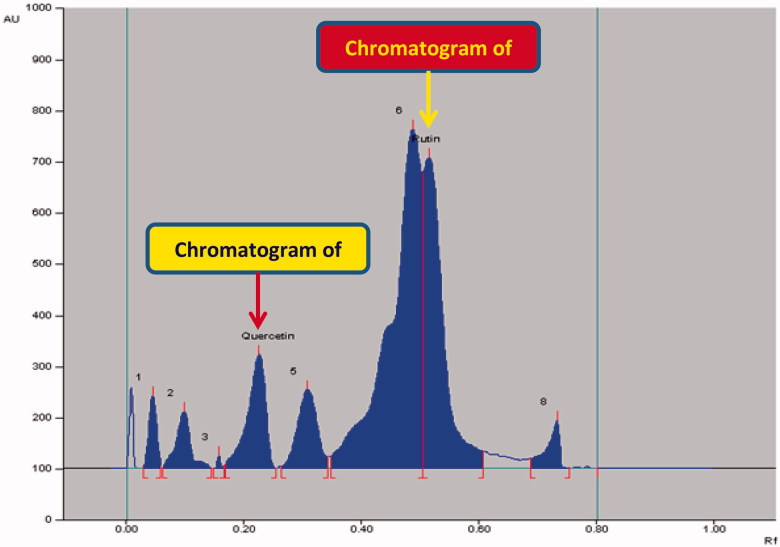
Chromatogram of GSEE (quercetin, spot 4, R_f_ = 0.23; rutin, spot 7, R_f_ = 0.52) at 360 nm; mobile phase, acetonitrile:water (4:6, v/v).

**Figure 6.**
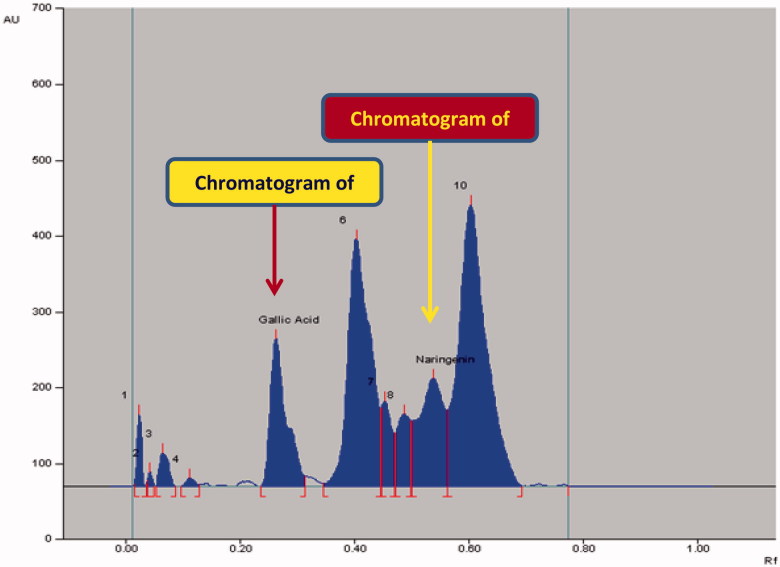
Chromatogram of GSEE (gallic acid, spot 5, R_f_ = 0.28; naringenin, spot 9, R_f_ = 0.56) at λ = 275 nm; mobile phase, toluene:ethyl acetate:formic acid (6:4:0.8, v/v/v).

The application of the proposed method was evaluated by applying this method for the quantitative analysis of rutin plus quercetin (Figure 5) and naringenin plus gallic acid (Figure 6) in GSEE. Notably, though the obtained peaks were near to each other in the two HPTLC methods, the corresponding bands were very clearly separated (Figures 3 and 4). The calculated area of all peaks (AU) after their integration was peak-1: 1613.8; peak-2: 2310.4; peak-3: 105.3; peak-4 (quercetin): 5775.5; peak-5: 4389.5; peak-6: 29403.8; peak-7 (rutin): 17508.5 and peak-8: 1672.0 (Figure 5) whereas it was peak-1: 614.6; peak-2: 112.1; peak-3: 578.5; peak-4: 128.6; peak-5 (gallic acid): 4564.8; peak-6: 10477.3); peak-7: 1707.8; peak-8: 1822.9; peak-9 (naringenin): 4819.5 and peak-10: 15116.4 (Figure 6). The quantified contents of rutin, quercetin, naringenin and gallic acid in GSEE were 2.42, 1.53, 0.14 and 7.01 μg/mg of the dry weight of GSEE. This is the first report, demonstrating simple, accurate and rapid NP- and RP-HPTLC methods developed for the simultaneous quantification of antiviral biomarkers rutin, quercetin, naringenin and gallic acid in *G. senegalensis*.

## Discussion

*Guiera senegalensis*, popularly known as ‘Cure all’ folk medicine in West and Central Africa, is used to treat various metabolic and infectious diseases (Aniagu et al. 2005; Diatta et al. 2007; Somboro et al. 2011; Suleiman 2015). Though the therapeutic potential *G. senegalensis* is widely recognized, most of them are still at a preliminary level that need to be evaluated by scientific rationale and detailed research. In this report, we have developed NP- and RP-HPTLC methods for the quantification of four biomarkers: rutin, quercetin, naringenin and gallic acid in GSEE showing antiviral efficacy against hepatitis B (Parvez et al. 2016).

Rutin is a flavonoid that belongs to the family of vitamin C2, and is abundant in many vegetables, fruits and cereals. Rutin is a well-known antioxidant, anti-inflammatory and anti-cancer natural compound (Deschner et al. 1993; Guardia et al. 2001; Yanga et al. 2008; Lin et al. 2009), and is sold commercially. Very recently, it has been demonstrated for promising antiviral efficacy against murine norovirus (MNV-1) *in vitro* (Chéron et al. 2015). In the present study, quantification of rutin (2.42 μg/mg) in the GSEE by RP-HPTLC method supports its possible role in the inhibition of HBV gene expressions and DNA replication.

Quercetin is a flavonol found in natural products, especially in apples and onions (Hertog et al. 1993). Quercetin is known to have multifaceted biological and therapeutic effects including antioxidative, anticancer, antimicrobial, anti-inflammatory, cardioprotective, and hepatoprotective activities (Harwood et al. 2007; Hernández-Ortega et al. 2012; D’Andrea et al. 2015). In addition, quercetin has *in vitro* antiviral activities against enveloped viruses such as meningovirus, *Herpes simplex* virus (HSV1), parainfluenza type 3, pseudorabies virus, respiratory syncytial virus, *Sindbis* virus (Mucsi 1984; Kaul et al. 1985; Vrijsen et al. 1988; Wleklik et al. 1988; Lamien et al. 2005; Choi et al. 2009; Chiow et al. 2016), including HBV (Cheng et al. 2015). Our quantitative analysis by validated RP-HPTLC method demonstrated quercetin (1.53 μg/mg) in the dry-weight of GSEE, therefore, strongly supports its anti-HBV activity.

Naringenin is a flavanone found mainly in citrus fruits and tomatoes. Naringenin has many pharmacological properties including hypolipidemic, anti-hypertensive, anti-inflammatory, antioxidant, anti-fibrotic, and hepatoprotective activities (Yen et al. 2009; Cho et al. 2011; Hermenean et al. 2014; Motawi et al. 2014; Chtourou et al. 2015). Interestingly, naringenien has been also reported for its antiviral potential against HCV through blocking the assembly of intracellular viral particles (Goldwasser et al. 2011). Our quantification of naringenin (0.14 μg/mg), though at a low level in SSEE by NP-HPTLC method indicates its possible role in inhibiting HBV life cycle.

Gallic acid is a phenolic compound obtained from plants, fruits and vegetables. Gallic acid and structurally related compounds possess many potential therapeutic properties including anti-cancer, anti-inflammatory and anti-microbial effects (Inoue et al. 1995; You & Park 2001; Ow & Stupans 2003; Kim 2007; Chen et al. 2009; Ji et al. 2009; Deng et al. 2014; Oyedeji et al. 2014; Xiaoyong & Luming 2014). In addition, gallic acid exhibited antiviral activities against enterovirus-71 (Choi et al. 2010), *Herpes simplex* virus type 1 (HSV-1), anti-human immunodeficiency virus (Kratz et al. 2008) and hepatitis C virus (HCV) (Govea-Salas et al. 2016). In our quantitative analysis by validated NP-HPTLC method, gallic acid (7.01 μg/mg) was estimated the most abundant biomarker in GSEE. Identification of gallic acid known for antiviral potential is in line with its recently reported anti-HBV activity.

As discussed above, the four biomarkers, rutin, quercetin, nargennin and gallic acid have antiviral potentials against a variety of biologically related but genetically RNA and DNA viruses. Of these, HSV, HIV and HBV are enveloped (coated) viruses but unlike HSV and HIV, HBV is a DNA virus. Notably, HBV uniquely replicates its DNA genome via an RNA intermediate through reverse-transcription similar to RNA viruses. Interestingly therefore, almost all potential nucleos(t)ide-based antiviral agents developed for HSV and HIV, have been effective against HBV. Moreover, HCV, an enveloped RNA virus, does not share the antiviral regimens of HBV (except, the cytokine interferon). Therefore, the effectiveness of rutin, quercetin, nargennin and gallic acid against these viruses could be explained by considering the common inhibitory mechanism either targeting viral envelopes or reverse-transcriptases. Nevertheless, addressing this issue is out of the scope of the present study. Notably, except rutin, quercetin, naringenin and gallic acid, we could not study the other antiviral biomrkers in GSEE due to some limitations. There is a very high possibility of presence of other potential biomarkers in *G. senegalensis* that needs further analysis.

## Conclusions

Our quantitative analysis of four antiviral biomarkers by the RP- and NP-HPTLC methods furnished gallic acid (7.01 μg/mg) the most abundant antiviral biomarker compared to rutin (2.42 μg/mg), quercetin (1.53 μg/mg) and naringenin (0.14 μg/mg) in *G. senegalensis* leaves. To the best of our knowledge, this is the first report demonstrating validation of simple, accurate and sensitive NP- and RP-HPTLC methods for the separation of different phytoconstituents and simultaneous quantification of antiviral biomarkers in *G. senegalensis*. In addition, our data scientifically endorses the traditional knowledge of *G. senegalensis* in folk medicine, including its anti-HBV activities. The developed methods could be therefore, employed in the standardization and quality-control of herbal preparations containing therapeutic biomarkers.
